# Hyaline fibromatosis syndrome: a case presenting with gingival enlargement as the only clinical manifestation and a report of two new mutations in the *ANTXR2* gene

**DOI:** 10.1186/s12903-021-01840-5

**Published:** 2021-10-09

**Authors:** Yiying Liu, Xin Zeng, Yi Ding, Yi Xu, Dingyu Duan

**Affiliations:** 1 State Key Laboratory of Oral Diseases, National Clinical Research Center for Oral Diseases, Department of Periodontology, West China Hospital of Stomatology, Sichuan University, No.14, Section 3, Renmin South Road, Chengdu, 610041 China; 2 State Key Laboratory of Oral Diseases, National Clinical Research Center for Oral Diseases, Chinese Academy of Medical Sciences Research Unit of Oral Carcinogenesis and Management, West China Hospital of Stomatology, Sichuan University, Chengdu, China

**Keywords:** Hyaline fibromatosis syndrome, Gingival enlargement, Differential diagnosis, Case report

## Abstract

**Background:**

Hyaline fibromatosis syndrome (HFS) is a rare autosomal recessive disorder caused by mutations in the gene for anthrax toxin receptor-2 (*ANTXR2*). The clinical features of HFS include skin thickening with nodules, papules and plaques, gingival enlargement, joint stiffness and contractures, and systemic manifestations. Notably, in all patients with HFS reported in the literature, gingival enlargement has never occurred alone.

**Case presentation:**

A case of a child with gingival enlargement as the only clinical manifestation, who was later diagnosed with HFS, is described. In this case, the absence of skin and joint lesions and other characteristic clinical presentations gave rise to a diagnostic problem. This uncommon condition was clinically indistinguishable from other diseases or conditions that presented with diffuse gingival enlargement. A definitive diagnosis of HFS was reached through genetic analysis. Trio whole exome sequencing revealed compound heterozygous mutations of *ANTXR2* in this patient and two new mutations were reported.

**Conclusions:**

The findings of this case serve as an important reminder to clinicians. When dental practitioners encounter gingival manifestations of HFS without accompanied skin or joint involvement, there is a need to pay attention to the differential diagnosis and increase awareness of HFS.

**Supplementary Information:**

The online version contains supplementary material available at 10.1186/s12903-021-01840-5.

## Background

Hyaline fibromatosis syndrome (HFS; MIM# 228600) is a rare autosomal recessive disorder of the connective tissue caused by mutations in the gene for anthrax toxin receptor-2 (*ANTXR2*), also known as capillary morphogenesis protein 2, located on chromosome 4q21 [1–3]. HFS is characterized by an abnormal deposition of hyaline-like material in the skin, mucosa, joints and internal organs. The clinical features of HFS include skin thickening with nodules, papules and plaques, gingival enlargement, painful joint stiffness and contractures, and occasional systemic involvement.

The severity of HFS varies between the mild form with limited skin involvement and the severe form with an earlier age of onset, visceral involvement, and early lethality [4–6]. A grading system of HFS was proposed by Nofal et al. [4] and modified by Denadai et al. [5], which classifies HFS into grade 1 or mild (skin and/or gingival involvement), grade 2 or moderate (joint and/or bone involvement), grade 3 or severe (internal organ involvement with or without clinical manifestations), and grade 4 or lethal (severe clinical decompensation).

Generally, most patients with HFS develop subcutaneous nodules and gingival enlargement. However, only a few patients reported in the literature presented with sole cutaneous and mucosal manifestations and were classified as having grade 1 or mild HFS. Importantly, all patients with grade 1 HFS reported had characteristic skin lesions [2–4, 7–14]. In this paper, we present a case of HFS with gingival enlargement as the initial and sole manifestation in the absence of subcutaneous nodules. This unique situation, which would lead to difficulties in the differential diagnosis between HFS and other diseases characterized by gingival fibromatosis, requires special attention among dental practitioners. In addition, two new mutations in the *ANTXR2* gene were reported.

### Case presentation

A 6-year-old boy had a history of gingival overgrowth, which had been present since the boy’s first tooth erupted at 6 months of age. The painless overgrowth of the gingiva progressed slowly causing no discomfort up to the age of 6 years, when the esthetic appearance also became intolerable. No other complaints were noted. The child was systemically healthy and was the only child of two healthy non-consanguineous parents. Similar findings or a history of such findings were not identified in either the parents or their relatives.

**Fig. 1:**
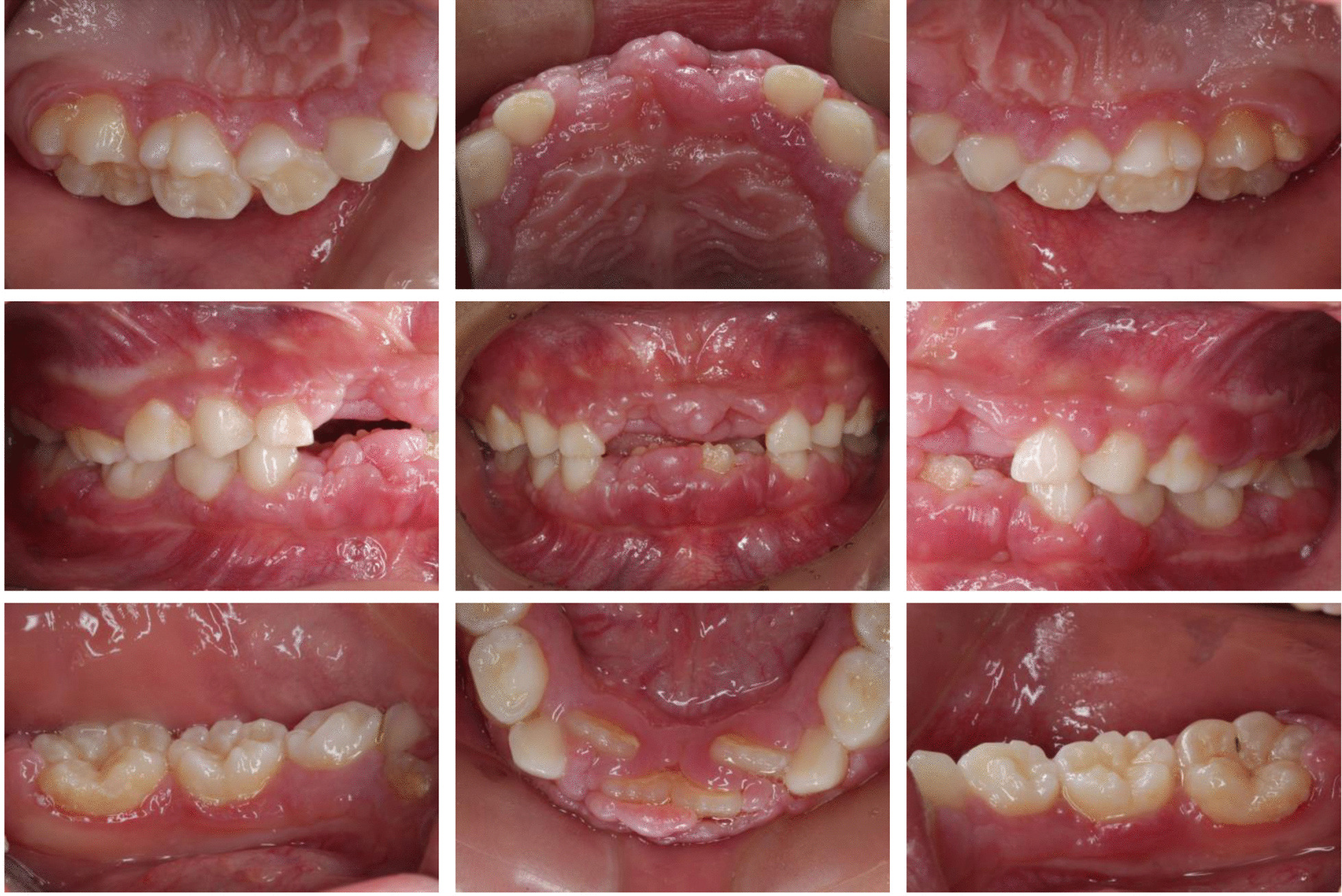
Intraoral view of the 6-year-old boy at the first appointment. Note the severe gingival enlargement

Oral cavity examination showed severe gingival enlargement involving both the mandibular and maxillary arches and was particularly prominent over the anterior regions (Fig. 1). The patient’s gingivae seemed to have slight redness and mild edema, with absence of normal stippling. The oral hygiene was poor due to the formation of pseudopockets containing large amounts of dental plaque and a small amount of calculus. As a result, the marginal gingiva was slightly inflamed. Apart from gingival enlargement, other mucosal lesions such as submucosal deposits or thickening, were not observed. Radiographical examination revealed no periodontal bone destruction.

**Fig. 2:**
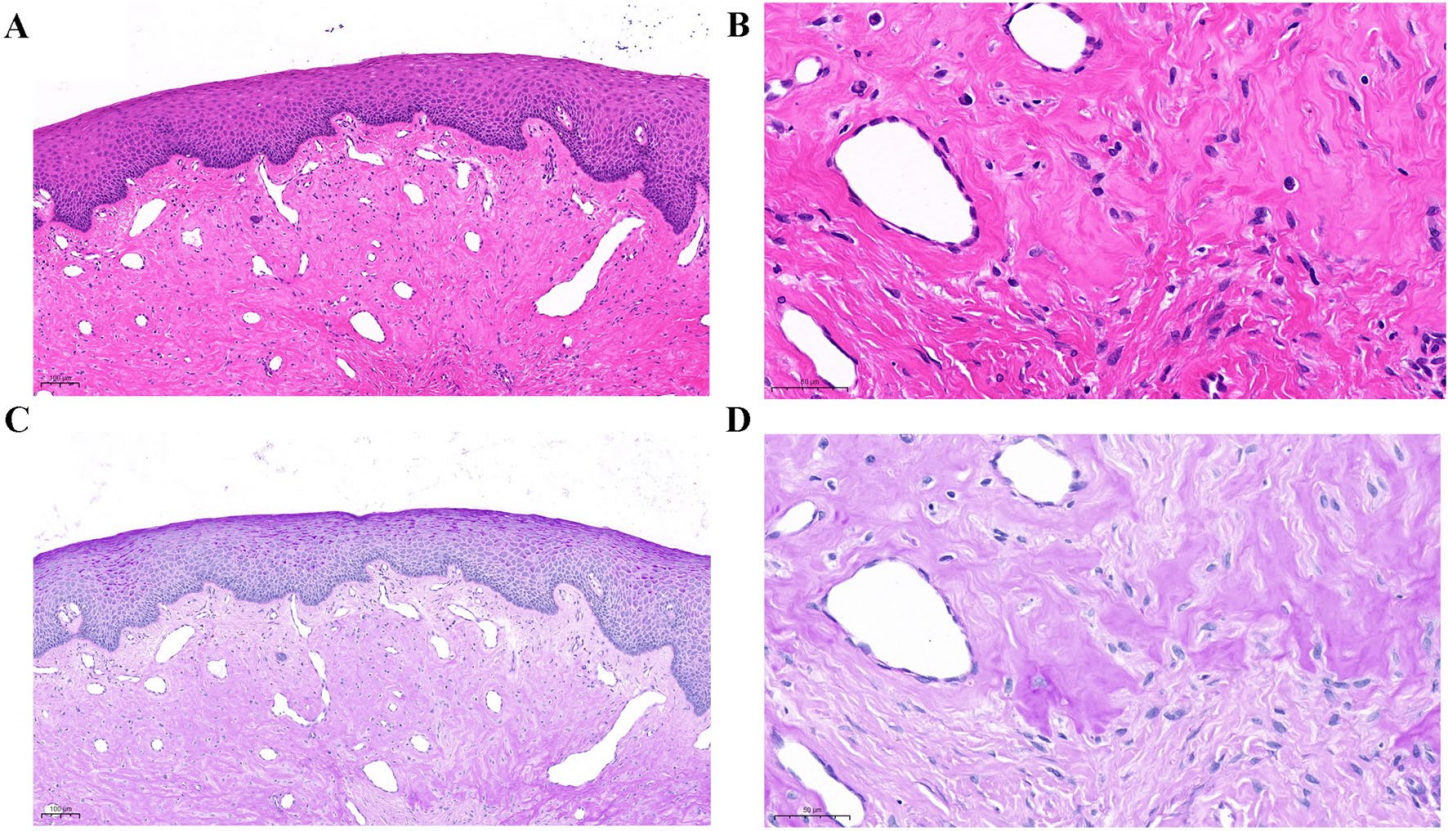
Histopathologic image from a gingiva biopsy showing (**A**, **B**) hyperplastic epithelium, mild inflammatory cell infiltration, proliferation of the capillary layer, and increase in fibrous tissue (hematoxylin–eosin stain), (**C**, **D**) deposits of an amorphous, homogeneous, and PAS-positive hyaline substance in the lamina propria and perivascular spaces (periodic acid Schiff stain)

The routine hematological and biochemical test results were unremarkable and initial phase therapy was administered. The gingival enlargement was not alleviated after the gingival inflammation was controlled. Gingivectomy was performed, and the wound healing was uneventful. Tissue specimens obtained during gingivectomy were sent for histopathologic examination. Hematoxylin–eosin (HE) staining revealed a hyperplastic epithelium, mild inflammatory cell infiltration, proliferation of the capillary layer, and an increase in the amount of fibrous tissue (Fig. 2A, B). This result did not indicate a definitive diagnosis. Meanwhile, recurrence of gingival enlargement occurred 1 week after the gingivectomy. Since this child presented with gingival enlargement as the only clinical manifestation and showed no other systemic involvement, the diagnosis proved difficult. Hence, genetic tests were performed.

**Fig. 3:**
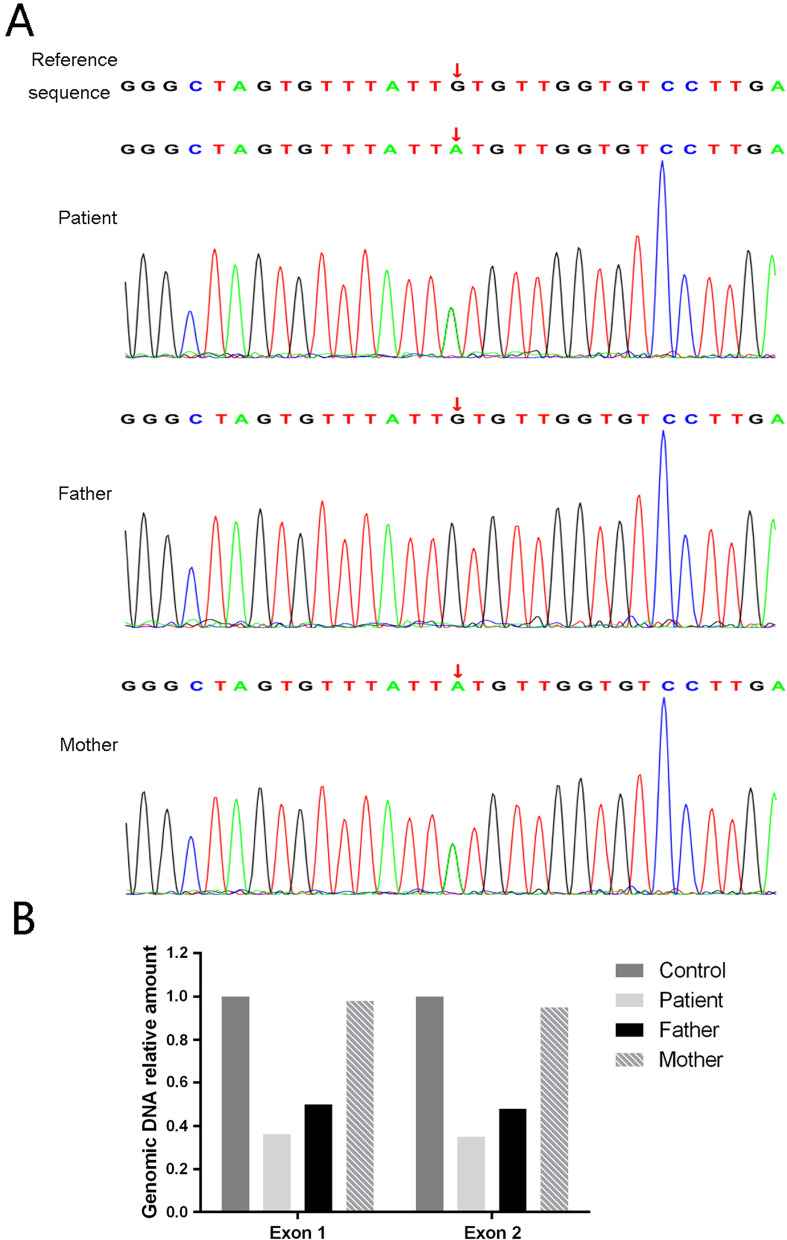
Mutation analyses for the anthrax toxin receptor 2 gene in the family members of the patient. **A** The c.524G > A mutation in exon 6 was observed in the patient and his mother. **B** Real-time polymerase chain reaction assay of the *ANTXR2* normalized by the human albumin gene showed the relative amounts of exons 1 and 2 in family members. The father and the patient had partial gene deletions involving both exons 1 and 2

Trio whole exome sequencing (WES) of DNA isolated from peripheral blood was performed at the Chigene Translational Medicine Research Center Co. Ltd. (Beijing, China). Detailed methods of genetic tests are provided in the supplementary information (Additional file 1). Genetic analysis revealed compound heterozygous mutations of *ANTXR2* in this patient: c.524G > A (p.Cys175Tyr) in exon 6 and loss of exons 1 and 2. The patient’s father had a heterozygous *ANTXR2* mutation, loss of exons 1 and 2, and his mother had a heterozygous *ANTXR2* mutation, c.524G > A (Fig. 3). The mutation in exon 6 of *ANTXR2* was further verified via Sanger sequencing, and the loss of exons 1 and 2 in *ANTXR2* was further verified by quantifying the copies of exons 1 and 2 via quantitative real-time polymerase chain reaction assays (Fig. 3).

Genetic analysis confirmed the diagnosis of HFS. Therefore, further physical, radiographic, and histopathologic examinations were performed after a literature review. No skin lesions or joint contractures were observed. Skeletal radiography showed no abnormalities of the bilateral distal humeri, radii, ulnae, carpal bones, metacarpals, distal femurs, tibiae, fibulae, metatarsi, and phalanges. The gingival-specimen sections were stained with periodic acid Schiff (PAS) and Congo red. Deposits of an amorphous, homogeneous, and PAS-positive hyaline substance were found in the lamina propria and perivascular spaces (Fig. 2C, D). The hyaline substance could not be stained with Congo red.

Based on the clinical presentation and genetic analysis results, the child was diagnosed with HFS of grade 1. A treatment plan, including regular periodontal debridement, oral hygiene motivation, and gingivectomy when necessary was proposed. As HFS is a progressive disease, and manifestations tend to be additive over time, the child was referred to a pediatrician for regular follow-up considering the possibility of systemic involvement in the future.

## Discussion and conclusions

Skin lesions such as subcutaneous nodules, papules, and plaques are the most common feature of HFS. These lesions typically occur in the scalp, ears, neck, face, hands, and feet. Additional features include gingival enlargement, joint stiffness and contractures, osteopenia, and osteoporosis. The severer form of HFS, which was previously referred as infantile systemic hyalinosis, shows internal organ involvement with or without systemic manifestations and can be lethal [5, 6]. The diagnosis of HFS is usually based on typical clinical manifestations combined with histopathologic characteristics, such as hyaline-like material in tissues. When HFS is suspected, sequencing of all exons and flanking sequences of the *ANTXR2* gene on chromosome 4q21 should be performed to genetically confirm the diagnosis..

When characteristic skin or joint lesions are present, HFS is not difficult to diagnose. However, given that gingival enlargement may be the initial and only manifestation in a case of HFS, and the hyaline-like material in gingiva may be hardly distinguishable from collagen fiber bundles in the HE stained section, as we reported in the present study, the differential diagnosis between HFS and other gingival fibromatosis diseases is both difficult and important.

Diseases that should be considered in the differential diagnosis of diffuse gingival enlargement are listed in Table 1. As the gingiva did not show much inflammation and the gingival enlargement was not relieved after periodontal debridement in this patient, the diagnosis of chronic gingivitis could be easily excluded. Moreover, drug-induced gingival enlargement, leukemia, vitamin C deficiency, and Crohn’s disease were not considered as candidate diagnoses because the medical history and blood tests of the child were both unremarkable. Among the genetic diseases, hereditary gingival fibromatosis was first considered because of the increase of fibrous tissue observed with HE staining. However, the gingiva of this patient was not as firm and dense as that of patients with hereditary gingival fibromatosis. Ultimately, we had to resort to genetic testing to help arrive at a diagnosis.

**Table 1:** Differential diagnoses of generalized gingival overgrowth in children

Disease	Features	Inheritance	Chromosomal region/gene locus
Gingivitis	The inflammatory response to a local irritant can lead to chronic hyperplastic gingivitis. Gingival enlargement is usually limited to areas affected by the irritant		
Drug-induced gingival overgrowth [21]	Intake of anticonvulsants, immunosuppressants, calcium channel blockers, or other drugs implicated in the occurrence of gingival overgrowth		
*Gingival overgrowth associated with systemic diseases*			
Leukemia [22]	Anemia, neutropenia, thrombocytopenia, easy fatigability, fever, bone pain, spontaneous gingival bleeding, gingival enlargement, petechial hemorrhages, mucosal pallor, herpetic infections, candidiasis, and oral ulceration		
Vitamin C deficiency [23]	Vitamin C deficiency is defined as a serum ascorbic acid level < 2 μg/mL. Clinical manifestations of scurvy include anemia, myalgia, bone pain, swelling, gingival enlargement, poor wound healing, and spontaneous bleeding		
Crohn’s disease [24]	Crohn’s disease is a chronic gastrointestinal inflammatory disease. Clinical manifestations include abdominal pain, diarrhea, nausea, vomiting, and weight loss. Oral manifestations include mucosal tags; swelling of the lips, cheeks, and gingiva; and cobblestone appearance of the mucosa		
*Genetic diseases and syndromes*			
Neurofibromatosis, type I (MIM 162200) [25]	Café-au-lait macules, axillary and inguinal freckling, neurofibromas, plexiform neurofibroma, optic pathway gliomas, sphenoid wing dysplasia or long-bone dysplasia, and Lisch nodules	Autosomal dominant	17q11.2
Hereditary gingival fibromatosis (MIM 135300, 605544, 609955 et al.) [26–28]	Benign, slow-growing, non-hemorrhagic, fibrous hyperplasia of the maxillary and mandibular gingiva	Autosomal dominant	2p22.1, 5q13-q22, 2p23.3-p22.3 et al
Congenital generalized hypertrichosis with or without gingival hyperplasia (MIM 135400) [29, 30]	Hypertrichosis, gingival hyperplasia, dysmorphic and coarse facies	Autosomal dominant, autosomal recessive	17q24.2-q24.3
Mucolipidosis (MIM 252500, 252600) [31]	Short stature, skeletal abnormalities, cardiomegaly, developmental delay, and gingival hyperplasia	Autosomal recessive	12q23.2
Winchester syndrome (MIM 277950) [32]	Generalized osteoporosis, multicentric osteolysis, and progressive joint destruction	Autosomal recessive	14q11.2
Zimmermann-Laband syndrome (MIM 135500, 616455, 618658) [33–35]	Gingival fibromatosis, hypo/aplastic nails and distal phalanges, hepatosplenomegaly, hypertrichosis, joint hypermobility, and abnormalities of the cartilage of the nose and/or ears	Autosomal dominant	1q32.2, 8p21.3, 1q21.3
Frank-ter Haar syndrome (MIM 249420) [36]	Thick skin, osteolysis, gingival hypertrophy, craniofacial anomalies, skeletal dysplasia, and cardiac defects	Autosomal recessive	5q35.1
Amelogenesis imperfecta, type IG (MIM 204690) [37]	Generalized thin hypoplastic or absent enamel in the primary and permanent teeth, pulp stones, delayed tooth eruption, root dilacerations of impacted teeth, gingival hyperplasia, and nephrocalcinosis	Autosomal recessive	17q24.2
Raine syndrome (MIM 259775) [38]	Generalized osteosclerosis, craniofacial dysplasia, thoracic hypoplasia, and gingival hyperplasia	Autosomal recessive	7p22.3
Gingival fibromatosis with progressive deafness (MIM 135550) [39]	Gingival fibromatosis associated with progressive neural hearing loss	Autosomal dominant	/
Gingival fibromatosis with distinctive facies (MIM 228560) [40]	Gingival fibromatosis, macrocephaly, bushy eyebrows, synophrys, hypertelorism, downslanting palpebral fissures, flattened nasal bridge, hypoplastic nares, cupid-bow mouth, and highly arched palate	Autosomal recessive	/
Ramon syndrome (MIM 266270) [41, 42]	Gingival fibromatosis, cherubism, short stature, mental deficiency, hypertrichosis, juvenile rheumatoid arthritis, and epilepsy	Autosomal recessive	/
Rutherfurd syndrome (MIM 180900) [43, 44]	Failure of eruption of teeth, gingival hyperplasia, dense corneal opacities	Autosomal dominant	/

This patient was diagnosed with grade 1 HFS according to the grading system of HFS [5]. Genetic analysis showed compound heterozygous mutations of *ANTXR2* in this child: c.524G > A in exon 6 and loss of exons 1 and 2. To the best of our knowledge, these two mutations have not been previously identified. Loss of exons 1 and 2 could result in deletion of 75 amino acids in the vWA domain and significant change in the protein sequence, while c.524G > A in exon 6 might result in amino acid change in the coding protein at position 175 (p.Cys175Tyr). According to the previous references [6, 15], missense mutations in the vWA domain, other missense mutation in exons 1–11, and mutations leading to premature stop codons (frameshift and splicing mutations) were associated with the severe form of HFS, while missense mutations in exons 13–17 were associated with the mild form of the disease. However, in this case, the genotype could not explain the mild phenotype of the patient. It appears that genotypes are not sufficient to account for all clinical variations. This phenomenon has also been observed in other case reports [3, 16, 17]. Therefore, in addition to the gene itself, there may be other factors, such as modifier genes and environmental elements, that can influence the HFS phenotype. Further research is required to elucidate this phenomenon.

To date, there is no effective treatment for HFS, and supportive care is the mainstay of treatment [6]. Lesions may require recurrent excision and tend to worsen during adolescence [5, 18]. Fortunately, some studies have shown the potential of personalized treatment such as proteasome-inhibitor administration [19] and therapies that inhibit the nonsense-mediated mRNA decay pathway [20], when the different molecular consequences of *ANTXR2* mutations are considered.

In conclusion, to the best of our knowledge, this was the first case of HFS solely presenting with gingival manifestations. It is possible that there have been similar cases which, however, remain undiagnosed, have not been reported. The findings of this case serve as an important reminder to clinicians. When dental practitioners encounter gingival manifestations of HFS without skin or joint involvement, there is a need to pay attention to the differential diagnosis and increase awareness of HFS.

## Supplementary Information

**Additional file 1.** Detailed methods of genetic tests for the patient.

## Data Availability

All data generated or analysed during this study are included in this published article.

## Abbreviations

HFS: Hyaline fibromatosis syndrome
*ANTXR2*: Anthrax toxin receptor-2
HE: Hematoxylin–eosin
WES: Whole exome sequencing
PAS: Periodic acid Schiff
