# Supplementary material for: Hyaline fibromatosis syndrome: a case presenting with gingival enlargement as the only clinical manifestation and a report of two new mutations in the ANTXR2 gene
Source: BMC Oral Health. 2021 Oct 9;21:508. doi: 10.1186/s12903-021-01840-5 (PMC8501544; doi:10.1186/s12903-021-01840-5)
Supplement: Supplementary file 1 — Additional file 1. Detailed methods of genetic tests for the patient. [file 12903_2021_1840_MOESM1_ESM.docx]

Supplementary Materials

The EDTA-treated peripheral blood were collected with informed consent of the patient and his parents, and genomic DNA was extracted using the Blood Genome Column Medium Extraction Kit (Kangweishiji, China) according to the manufactural instructions. Next, trio whole exome sequencing (WES) was performed at the Chigene Translational Medicine Research Center Co. Ltd. (Beijing, China). In brief, protein-coding exome enrichment was performed using xGen Exome Research Panel v1.0 (IDT, Iowa, USA). The high-throughput sequencing was performed by Illumina NovaSeq 6000 series sequencer (PE150), and not less than 99% of target sequence were sequenced. The paired-end reads were performed using Burrows-Wheeler Aligner (BWA) to the Ensemble GRCh37/hg19 reference genome. Base quality score recalibration together with SNP and short indel calling was conducted using GATK. Variants annotation and pathogenicity prediction were performed through the online system independently developed by Chigene ([www.chigene.org](http://www.chigene.org)). This system was used to annotate database-based minor allele frequencies (MAFs), and ACMG practice guideline-based pathogenicity of every yielded gene variant, and also provide a serial software packages for conservative analysis and protein product structure prediction.

The mutation in exon 6 of *ANTXR2* was further verified via Sanger sequencing. PCR amplification of the exon 6 (E6) was performed using KAPA2G Robust DNA polymerase (Kapa Biosystems, Darmstadt, Germany). Primer sequences used are detailed in Table S1. Gel purification was used to purify the PCR products according to the manufacturer's instructions (SanPrep Column DNA Gel Extraction kit, Sangon Biotech Co., Ltd., Shanghai, China). Sanger sequencing was performed using an ABI 3730 automatic sequencer (Thermo Fisher Scientific., Inc., Waltham, MA, USA). Finally, mutation analysis was performed through DNASTAR software (DNASTAR, Inc., Madison, WI, USA).

The loss of exon 1 and 2 (E1 and E2) in *ANTXR2* was further verified by quantifying the copies of exon 1 and 2 via quantitative real-time polymerase chain reaction assays using TB Green™ Premix Ex Taq™ Ⅱ (Tli RNaseH Plus; Takara Bio, Dalian, China) according to the manufacturer's instructions. Primer sequences used are listed in Table S1. The results were normalized by the human albumin gene.

Table S1. Primers designed for PCR and real-time PCR.

| Name | Primer sequence (5’→3’) | Product （bp） | Annealing temperature |
| --- | --- | --- | --- |
| E6 | F: TAATTTCTGTAAATAAGGGCTA  R: AAAATCTTTAACAATCGACCA | 1450 | 60 |
| Albumin | F: AGTGCACTTGTTGAGCTCGTG  R: GCAAAGCAGGTCTCCTTATCG | 128 | 60 |
| E1 | F: CTGCGAGCGGGAGGGAGTCT  R: CGGGACCGCTGAGCACCAA | 120 | 65 |
| E2 | F: GCCCCTATGTTCGTGGCATTGA  R: TCTCTCCGCAAGTTGCTGTACG | 162 | 65 |
